# Mast cell-derived exosomes and claudin regulation in ulcerative colitis: emerging insights and therapeutic potential

**DOI:** 10.1039/d5na00707k

**Published:** 2025-09-10

**Authors:** Shao-han Li, Hao-ming Xu, Hong-li Huang, Yong-jian Zhou

**Affiliations:** a Department of Gastroenterology and Hepatology, Second Affiliated Hospital, School of Medicine, South China University of Technology No. 1 Panfu Road Guangzhou 510180 China; b Department of Gastroenterology and Hepatology, Guangzhou Digestive Disease Center, Guangzhou First People's Hospital No. 1 Panfu Road Guangzhou 510180 China

## Abstract

Ulcerative colitis (UC) is a chronic inflammatory condition marked by immune dysfunction and disruption of the intestinal epithelial barrier, in which mast cells play a significant role through the release of inflammatory mediators. Recent advances suggest that mast cell-derived exosomes and intraluminal vesicles (MC-EXOs and MC-ILVs) may contribute to disease pathogenesis by modulating epithelial tight junction proteins, particularly members of the Claudin family. Notably, transcriptomic analyses indicate that CLDN23, a gene encoding Claudin-23, is downregulated in active UC. Exosomes are emerging as key players in intercellular communication, capable of delivering functional microRNAs and proteins that influence intestinal permeability and immune cell behaviour. This mini-review summarizes current evidence on the interaction between mast cell-derived vesicles and intestinal epithelial cells, focusing on their regulatory role in Claudin expression and immune signalling pathways. Understanding these mechanisms may inform the development of exosome-based biomarkers and therapeutic strategies for UC.

## Abstract 2

Ulcerative colitis (UC) is a chronic inflammatory condition marked by immune dysfunction and disruption of the intestinal epithelial barrier, in which mast cells play a significant role through the release of inflammatory mediators.

## Graphical abstract

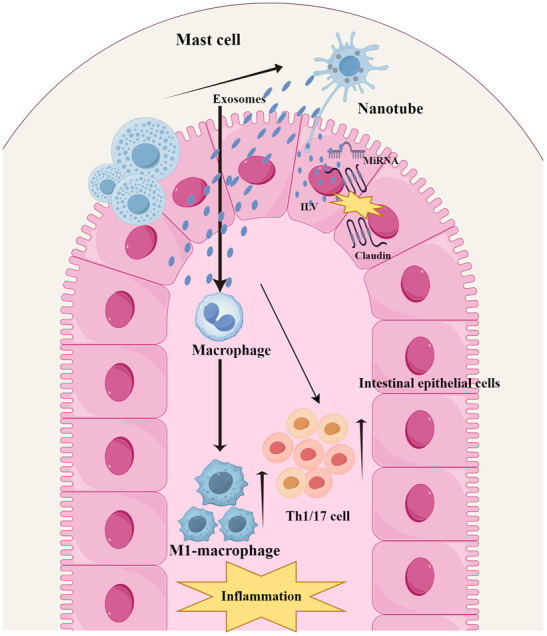

## 1. Introduction

Ulcerative colitis (UC) is an inflammatory bowel disease (IBD) of unknown etiology. It is characterized by multiple causative factors and potentially has lifelong consequences, which significantly impact the patients' quality of life.^1–3^ Evidence from current research suggests that UC pathogenesis is primarily related to intestinal immune imbalance, intestinal flora dysbiosis, and other genetic factors.^4^ The clinical treatment of UC primarily focuses on controlling inflammation and relieving symptoms. Recent findings have revealed that exosomes are stable, compatible, and safe nano-scale delivery vehicles within the human body and do not exhibit immune-activating properties. Designing methods for delivering certain drug treatments has become a popular research topic.^5,6^ However, when secreted by certain cells, exosomes not only serve as a structural shell but also exhibit diverse functions.

Exosomes are small vesicles with a diameter of 30–200 nm that can be secreted by various cells into the extracellular space. They contain various bioactive components, including nucleic acids, proteins, and lipids.^7–9^ Intraluminal vesicles (ILVs) represent the pre-secretory state of exosomes and can form multivesicular bodies intracellularly.^10^ Exosomes, naturally secreted nanoscale vesicles derived from cells, are ubiquitous and perform diverse functions. For example, exosomes derived from oxidized low-density lipoprotein-induced macrophages are enriched in miRNA-146a. This causes the accumulation of lipid-containing macrophages in the vascular wall and leads to atherosclerosis.^11^ Mesenchymal stem cell-derived exosomes can enhance the skin barrier and suppress the release of inflammatory mediators following skin injury.^12,13^ Furthermore, exosomes not only function as natural nanoparticles but also undergo complex engineering modifications. For instance, dendritic cell-derived chimeric exosomes encapsulating toyocamycin enable targeted drug delivery to tumors, and aptamer-modified macrophage-derived exosomes facilitate bone injury repair.^14,15^

Mast cells (MCs) are widely distributed in various tissues, such as blood vessels, skin, nerves, and the intestinal mucosa, among other tissues.^16^ Mast cell-derived exosomes (MC-EXOs) have garnered widespread attention in disease pathogenesis.^17–19^ Furthermore, previous studies have reported that Argonaute 2 (Ago2) protein influences both exosome secretion and miRNA selection within exosomes.^20,21^

Recent studies have demonstrated that tight junction (TJ) proteins, such as the Claudin family of proteins, play a crucial role in maintaining intestinal epithelial barrier function in UC.^22^ Adjacent cells in the intestinal epithelium are connected by TJs. The Claudin proteins, as important components, bind to the cytoskeletal domains of the cellular scaffolds such as ZO-1 and ZO-2, forming a connective network that protects the intestinal barrier.^23^ Claudin proteins are characterized by four transmembrane domains with a long intracellular C-terminus (PDZ-binding domain) and a shorter N-terminus. They also contain two extracellular loops: the larger ECL1 and the smaller ECL2. These structures are encoded by the Claudin gene family, which comprises 27 members in mammals.^24,25^ For instance, Claudin-3 is expressed in surface intestinal epithelial cells (IECs) and crypt enterocytes. The dysregulation of Claudin-3 expression can contribute to IBD development.^26^ Claudin-4 and Claudin-8 work synergistically to form anion channels, forming TJs in goblet cells. Their dysregulation can cause UC. Generally, the expression of most Claudin proteins is downregulated in IBD, with the notable exception of Claudin-2 expression, which is upregulated.^27–31^ Claudin-8, as a downstream target, can be influenced by IL-23 and CREBHKO, which can alter intestinal permeability and inflammation.^32,33^

## 2. The potential communication between vesicles and intestinal epithelium in ulcerative colitis

**Fig. 1:**
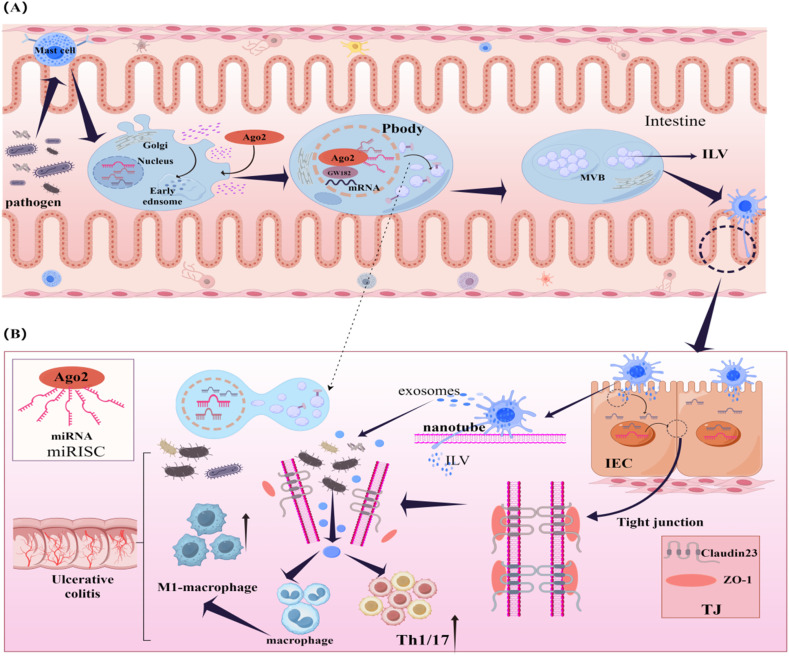
(A) Mast cell synthesizing intraluminal vesicles in the exocytosis endosomal pathway. To stabilize the translocated miRNA, the extracellular Argonaute 2 protein binds to the miRNA 5′ end, A/U region. It carries miRNA to form the miRNA-induced silencing complex. This complex combines GW182 and untranslated mRNAs to form a processing body that inhibits miRNA expression. Following this, degraded Argonaute 2 protein, mRNA, and GW182 mature into intraluminal vesicles. (B) Mast cell nanotubes delivering intraluminal vesicles to intestinal epithelial cells, as well as secreting exosomes involved in the regulation of macrophage and T cell subsets.

We hypothesize that MC-ILV may influence Claudin-23 expression, thereby contributing to UC pathogenesis. Mast cells generate vesicles *via* the endosomal pathway and establish contact with intestinal epithelial cells through their derived nanotubes, delivering intraluminal vesicles to epithelial cells. This process may reduce Claudin-23 protein distribution in the colon, decrease ZO-1 expression, polarize macrophages towards the M1 phenotype, and regulate Th1/Th17 cell numbers. These processes lead to altered intestinal permeability and diminished tolerance to immune or non-immune stimuli, ultimately resulting in persistent inflammation and the development of UC. Therefore, inhibiting MC-ILV synthesis represents a potential therapeutic approach for UC (Fig. 1). However, the speculative hypotheses are derived from secondary analyses of bioinformatics data and literature support. This hypothesis requires further validation.

## 3. Assessment

**Fig. 2:**
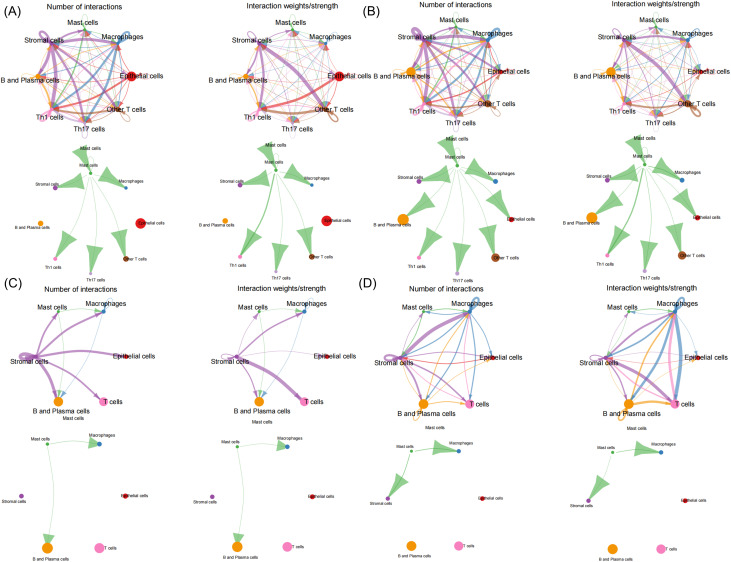
Re-analysis of the public data from GSE214695 to investigate cell to cell communication. (A) Total interaction number and strength between all cell types in the HC group and interaction number and strength specifically between mast cells and other cell types. (B) All pathways communication (number and weights) specifically between other cell populations and mast cells. (C) The number and intensity of ECM-receptor communication between mast cells and individual cells in the UC group. (D) The comparison of secreted signaling interaction strength and number between mast cells and distinct cell populations.

**Fig. 3:**
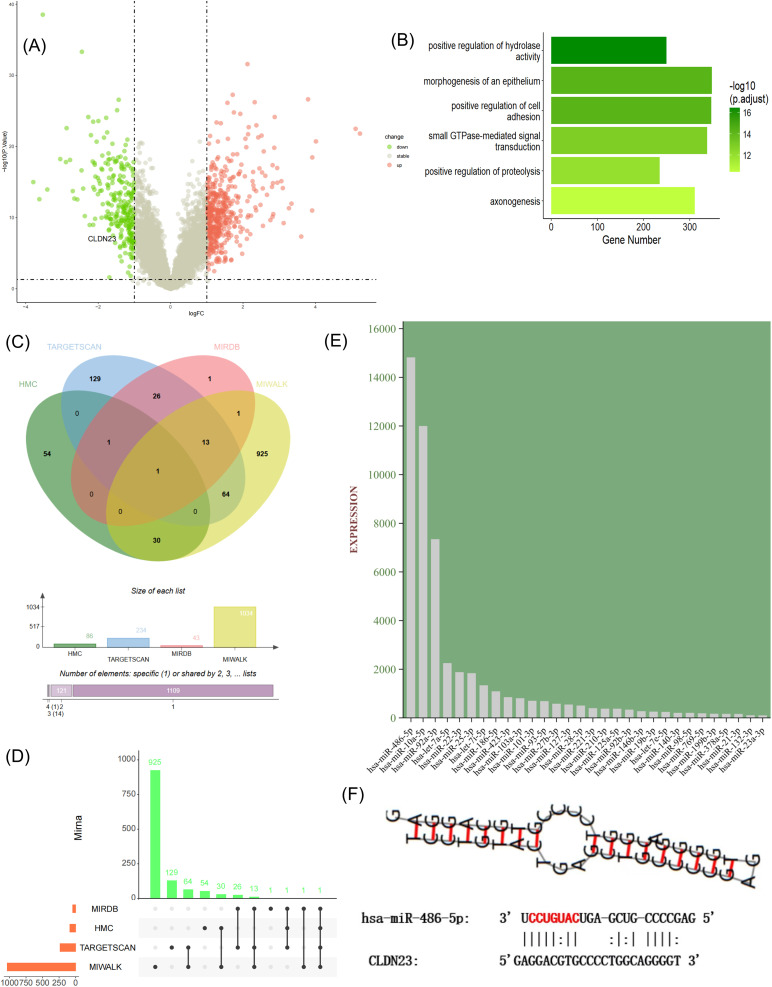
Re-analysis of the GSE87466 dataset to assess potential targets binding to CLDN23. (A) Differential gene analysis showing 267 downregulated genes, including CLDN23 (log Fc = −1.11345800, *P* < 0.05). (B) GO enrichment analysis of differentially expressed genes. (C and D) Overlap of HMC-miRNA expression types from the EVmiRNA database with TARGETSCAN, MIRDB and MIWALK databases. (E). Expression levels of CLDN23-targeting miRNAs within MC-EXOs. (F) Predicted binding sites between miRNA-486-5p and CLDN23.

From the GEO database, we re-analyzed high-throughput single-cell sequencing data (GSE214695) that included 6 healthy colon tissues and 6 colon tissue samples from active UC patients. Through t-SNE dimensionality reduction clustering and annotation, cells from healthy control (HC) and ulcerative colitis (UC) groups were classified into intestinal epithelial cells, T cells (including Th1, Th17 cells), macrophages, B cells and plasma cells, mesenchymal cells, and MCs (Fig S1A and C). Marker genes for each cell type are displayed in Fig. S1C. Notably, among these identified cell types, proportions analysis revealed a decreased MC percentage in the UC group (Fig. S1B). Previous studies report that activated MCs are more prevalent than their resting counterparts in UC. However, these activated cells undergo accelerated apoptosis after releasing proteases, which may account for the observed reduction in MC numbers within the UC group.^34^ Cell–cell communication analysis revealed close MC interaction with macrophages and Th1 cells in both HC and UC groups. In UC samples, MCs exhibited specific interactions with intestinal epithelial cells, implying direct MCs-epithelial contact (Fig. 2). Based on mast cell positioning in the intestine, they are mainly classified into two types: the lamina propria and epithelial types. The specific function of these cells remains unclear. However, recent research discovered that intraepithelial MCs regulate anti-helminth type 2 immunity *via* protease delivery through nanotube lumens, a key mechanism in MC-epithelial crosstalk.^35^ Reanalysis of the data serves as an exemple, suggesting that MC-EXOs or MC-ILVs may be transported to epithelial cells through nanotubes to modulate the epithelial barrier function in UC. Additionally, the secondary analysis revealed a significant downregulation of CLDN23 expression in UC and identified miRNAs that potentially target CLDN23 (Fig. 3). Past research has demonstrated that IECs expressing CLDN23 recruit CLDN3 and CLDN4, forming *cis*-interacting complexes that synergistically enhance the intestinal barrier function as contributors to tight junction assembly.^36^ Furthermore, UC is an autoimmune disease associated with pro-inflammatory M1 macrophages. Lu *et al.* found upregulated miRNA-21a-5p in M1 macrophage-derived exosomes from DSS-induced UC mice. This miRNA targets E-cadherin mRNA, inhibiting its translation and activation. Then, Th2 cells, through the miRNA-21a-5p/E-cadherin/KLRG1/GATA-3 pathway, aggravate DSS-induced colitis.^37^ Collectively, these previous literature reports motivated us to investigate unexplored connections between exosomes, UC pathogenesis, and Claudin proteins. Despite support from the cited literature, our perspective still requires rigorous experimental studies and validation in clinical cohorts to ensure its robustness.

## 4. Claudin underexpression contributes to UC development

Abnormalities in Claudin proteins have been implicated in damage to the intestinal mucosa in patients with UC. Xiao *et al.* demonstrated that matrix metalloproteinase 7 (MMP7) was overexpressed in the colon of patients with UC. MMP7 can cleave Claudin-7, compromising the protection provided by the intestinal barrier.^38^ Claudins play both barrier and non-barrier roles. Their functions include forming paracellular channels, facilitating signal transduction, regulating cell differentiation and proliferation, and maintaining cell membrane integrity.^39^ For instance, Claudin-12 promotes cancer cell migration and proliferation, while Claudin-5 restricts endothelial cell movement to enhance adhesion.^40,41^ Channels formed by Claudin proteins exhibit selective permeability. In the IECs, the overexpression of CLDN23 significantly reduces permeability to cations (Na^+^, Li^+^) and anions (Cl^−^).^36^ The dysregulation of Claudin-2 expression in UC primarily causes diarrhea by increasing the leakage of ions, solutes, and water from blood vessels through paracellular channels. In previous studies, patients with Crohn's disease exhibited higher expression of pore-forming Claudin-2 but lower expression of barrier-enhancing Claudin-3 and Claudin-4.^27^

Under normal conditions, the oxygen concentration gradient in the intestinal epithelium induces “physiological hypoxia,” which activates hypoxia-inducible factor 1 (HIF-1) to sustain the normal barrier. Findings from previous studies have demonstrated that the barrier effect in HIF-1β-deficient colonic epithelium is weakened in response to the absence of Claudin-1 expression. Conversely, Claudin-1 expression can improve barrier protection.^42^

Claudin-4, in conjunction with Claudin-8, can form both a sodium channel and a protective barrier. Hou *et al.* discovered that Claudin-8 binds Claudin-4 with high affinity in renal collecting tubules.^43^ In a porcine reproductive and respiratory syndrome virus infection model, Sun *et al.* found that Claudin-8 and Claudin-4 dysregulation in pulmonary microvascular endothelial cells increased vascular endothelium permeability.^44^ Additionally, a study on a human colon cancer HT-29-B6 cell model revealed that TGFβ-1 enhances barrier function by upregulating Claudin-4 expression through Smad-4-dependent transcription.^45^

Claudin-5 is expressed in both vascular endothelial cells and intestines. Using a DSS mouse model, Wang *et al.* verified that Claudin-5 is a target of the IL-21-mediated inflammatory cascade. Claudin-5 is positioned downstream of miRNA-423-5p in the IL-21/miRNA-423-5p/Claudin-5 pathway. Then, miRNA-423-5p binds to the 3′UTR sequence of Claudin-5, blocking its expression, damaging the barrier, and inducing UC. The expression of Claudin-1, Claudin-8, and ZO-1 was also inhibited in this model.^22^

**Fig. 4:**
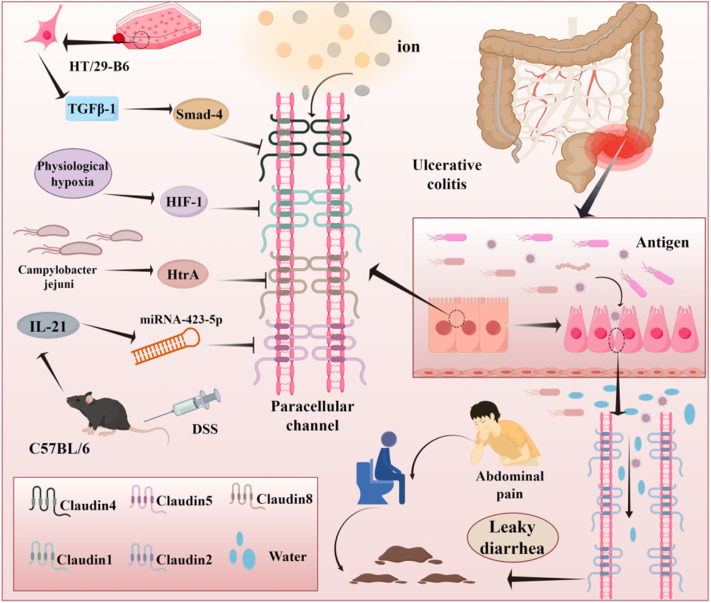
The role of different pathways in regulating claudin proteins. Typically, several factors can trigger the downregulation of Claudin protein, such as the TGFβ-1/Smad-4 pathway, HIF-1, HtrA, and miRNA. However, Claudin-2 shows great improvement in UC, which can lead to leaky diarrhea.

Infection is another focal point in investigations on UC pathogenesis. *Campylobacter jejuni* infection results in the secretion of high temperature requirement A protein, which cleaves Claudin-8 at the N-terminal cleavage site at position A58-N59. This allows pathogens to invade the paracellular space, triggering an intestinal inflammatory immune response^46^ (Fig. 4).

Claudin family members typically serve dual roles in barrier formation and channel regulation, with individual members (*e.g.*, CLDN5) exhibiting context-dependent functions. While research on barrier disruption in UC has extensively documented altered permeability phenotypes, the cascade regulatory effects mediated by dynamic TJ complexes remain poorly explored. Although key regulatory pathways contributing to UC development have been identified, how they in a coordinated or antagonistic manner target specific Claudin combinations to modulate barrier integrity and channel activity is unclear, highlighting the need for an integrated molecular model.

## 5. Exosomes are involved in UC regulation

Exosomes derived from various sources have been observed to either mitigate or contribute to UC development. On the protective side, exosomes derived from IECs (IECs-EXOs) maintain barrier integrity and activate the immunosuppressive function of Treg cells in the intestinal mucosa. IECs-EXOs contain annexin A1, which binds the IEC formyl peptide receptor to mediate the repair of damaged mucosa.^47,48^ Conversely, exosomes can be potent drivers of inflammation and barrier disruption in UC. Their cargo is highly diverse and context-dependent. For instance, exosomal DNA, whether surface-bound on small vesicles or encapsulated within larger ones, can act as a damage-associated molecular pattern. After being taken up, it can engage Toll-like receptors, triggering pro-inflammatory NF-κB signaling and exacerbating colitis, highlighting a potential link between circulating nucleic acids and IBD pathogenesis.^49,50^

Furthermore, exosomes are intimately linked to inflammasome activation. The NLRP3 inflammasome is involved in pyroptosis and mediates IL-1β/IL-18 activation in Th1/Th17 cells to damage the barrier.^51^ Recent studies have proposed that an exosome/NLRP3 inflammasome inflammatory cascade, in which non-stem cells derived exosomes may act directly on the NLRP3 inflammasome to induce caspase-1 cleavage and release, is involved in UC pathogenesis.^52^ Notably, the inflammatory effects of IL-1β can be sustained and amplified through exosome-dependent packaging. Gasdermin D (GSDMD) is considered to be involved in non-pyroptotic inflammatory action through IL-1β-dependent GSDMD release. Researchers found an increase in the GSDMD protein levels in IECs present in the inflamed regions of the mouse colon in a murine model of DSS-induced colitis. They demonstrated that IECs-EXOs release the GSDMD/NEDD4/caspase-8/IL-1β complex and identified a novel and potentially targetable inflammatory amplification loop.^53^

**Fig. 5:**
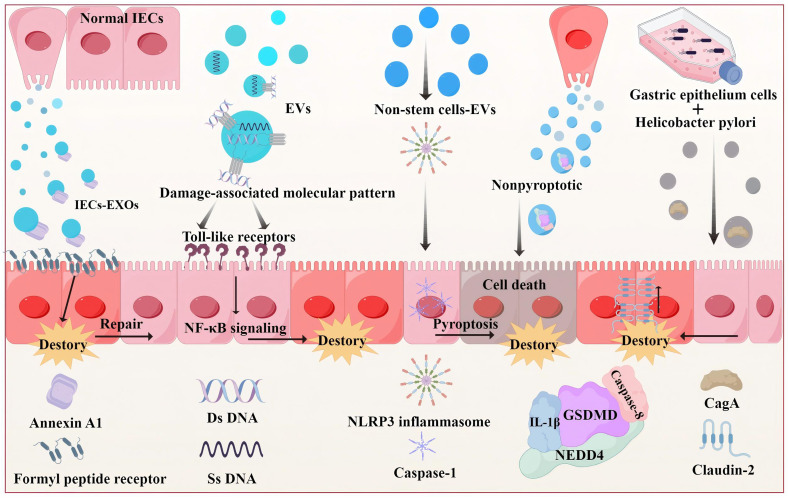
Exosomes from diverse sources mediating both repair and injury processes in the IECs through pyroptotic and non-pyroptotic mechanisms.

Perhaps most intriguingly, exosomes facilitate pathological communication beyond the local intestinal environment. The concept of “long-distance infection” may be exemplified by studies on *Helicobacter pylori*. Guo *et al.* demonstrated that gastric tissue-colonizing *H. pylori* secrete cytotoxin-associated gene A protein (CagA), which could exacerbate colitis in a DSS-treated mouse model. They found that CagA was encapsulated in human gastric epithelium cell derived exosomes and subsequently captured by the colonic epithelium. CagA activated CDX-2, which disrupted the integrity of the intestinal mucosal barrier and aggravated colitis. The activation of CDX2 promoted Claudin-2 expression and was accompanied by a loss of ZO-1 expression^54^ (Fig. 5).

In summary, exosomes exhibit dual regulatory roles in UC, influencing mucosal protection, inflammation amplification, and distant organ damage. Validation of the GSDMD-mediated non-pyroptotic pathway and remote infection theory in human UC samples remains essential. Targeting exosome packaging and release thus represents a promising therapeutic strategy. However, the dose–response relationship of exosomes within the inflammatory milieu of UC is poorly defined. While current purification strategies enhance the detection of exosome-mediated effects *in vitro*, they may not accurately reflect their pathophysiological relevance *in vivo*, where complex confounding factors significantly modulate outcomes.

## 6. Interaction between exosomes and claudin proteins

Exosomes secreted by various cells can circulate in the bloodstream, affecting multiple tissue sites and targeting different Claudin proteins. In previous studies on the mechanism underlying colorectal cancer metastasis, exosomes secreted by cancer cells were found to be transferred *via* blood flow and could alter blood vessel permeability. Liu *et al.* used epithelial–mesenchymal transition (EMT) HCT116 cancer cells co-cultured with human umbilical vein endothelial cells as recipient cells. They observed that the level of EMT-derived exosomes carrying miRNA-29a was prompted in the recipient cells. The miRNA-29a targets the transcription factor KLF4 through the EMT cancer cell-EXO/miRNA-29a/KLF4 pathway. This interaction destabilizes the vascular endothelial barrier by disrupting the connectivity between Claudin-5, occludin, and ZO-1, increasing the permeability of monolayer endothelial cells, thereby facilitating cancer cell metastasis.^55^

In addition to the effects of exosomes on them, Claudin proteins also influence the biogenesis of exosomes. Claudin-7 plays a role in TJs. Palmitoylated Claudin-7 constitutes glycolipid-enriched membrane domains. Cancer-initiating cell micro-vesicles with a recognized oncogenic role are used in an assay to compare the effects of palmitoylated and non-palmitoylated Claudin-7 on vesicle recruitment. The study showed that non-palmitoylated Claudin-7 was more conducive to vesicle assembly and that vesicle recycling and restoration of miRNA action were both dependent on non-palmitoylated Claudin-7. In contrast, palmitoylated Claudin-7 primarily facilitated the recruitment of glycolipid-enriched membrane domain-associated proteins.^56^ Ikeda *et al.* explored biomarkers for cholangiocarcinoma (CCA) and identified Claudin-3 protein in bile-derived extracellular vesicles. Using the chelating agent EDEG, high-purity extracellular vesicles were extracted from patients with CCA. Four proteins, namely Claudin-3, LARS, FAF2, and RAB20, which are potential markers, were analyzed using ELISA. They found that Claudin-3 expression was significantly elevated and proposed that it could be the most valuable potential diagnostic marker in CCA.^57^

These findings reveal an interplay between exosomes and TJs, suggesting an alternative pathway for exosome biogenesis. However, it remains unclear whether the context-specific targeting selectivity of Claudin proteins by exosomes exhibits tissue specificity. The diagnostic sensitivity and specificity of exosomes as biomarkers require validation in larger cohorts. In the future, studies need to integrate intravital imaging and spatial transcriptomic analyses to elucidate the spatiotemporal dynamics of exosome-Claudin interactions within disease microenvironments.

## 7. Exosomes secreted by MCs

Recent studies reveal that the exocytosis process of MC secretory granules influences exosome release. Omari *et al.* demonstrated that granule-plasma membrane fusion is mediated by CD63-positive, LC3 late endosomes. These endosomes form through Rab5-regulated internalization of the plasma membrane. Furthermore, the balance of exosome release is regulated by either the fusion of granules into large secretory vesicles or the fission of multigranular aggregates.^58^

MC-EXOs mediate ferroptosis in target cells. Fang *et al.* demonstrated that in a murine acute respiratory distress syndrome model, MC-EXOs were internalized by human bronchial epithelial cells, which were deficient in miR-774. This uptake suppressed GPX4 expression while elevating ACSL4 and ALOX15 levels, inducing ferroptosis and inflammation. Conversely, the analysis of blood-derived exosomes from ARDS patients revealed a significant reduction in miR-744 levels, suggesting its potential utility as a diagnostic biomarker.^59^ MC-EXOs also participate in cellular processes as functional complexes. Li *et al.* showed that the surfaces of exosomes secreted by bone marrow-derived mast cells express OX40L and CD63 molecules. Then, OX40L linked to OX40, which enabled the differentiation of CD4-positive T cells into Th2 cells.^60^ Wang *et al.* studied preeclampsia and found that miR181a-5p in MC-EXOs targeted the YY1/MMP-9 pathway and regulated the MMP-9 promoter to affect trophoblast activity.^19^ Nevertheless, while functional miRNAs and mRNAs are detected, further verification is required to confirm whether all identified transcripts undergo translation and functional expression. Although exosome biogenesis pathways are increasingly well-characterized, significant gaps persist in understanding their release kinetics, extracellular dissemination, and cellular uptake mechanisms (Table 1).

**Table 1:** Review of previous studies on miRNA and its binding targets

Micro RNAs	Targets	Conclusion	Year	Reference
miRNA-21a-5p	E-cadherin/KLRG1/GATA3	Unfavorable	2021	37
miRNA-423-5p	IL-21/Claudin-5	Unfavorable	2020	22
miRNA-29a	KLF4/ZO-1/Claudin-5	Unfavorable	2023	55
miRNA-774	GPX4	Beneficial	2024	59
miRNA-181-5p	YY1/MMP-9	Beneficial	2022	19
miRNA-214-3p	PI3K/AKT/mTOR	Beneficial	2024	61
miRNA-590-3p	LATS1/YAP/β-catenin	Beneficial	2021	62
miRNA-223	Claudin-8	Unfavorable	2016, 2020	32 and 63

## 8. Discussion and conclusion

Previous studies have shown that exosomes can be secreted with the endosomal sorting complexes required for transport (ESCRT), which comprises three components: ESCRT-I, ESCRT-II, and ESCRT-III. Among them, ESCRT-III can induce the deformation of the plasma membrane, facilitating the release of exosomes from plasma membrane buds.^64^ In recent studies, exosomes have been categorized into classical (positive for CD81, CD63, and CD9) and non-classical types based on their surface molecular phenotypes.^65^ Argonaute proteins play a supporting role in exosome release as well as in miRNA processing. These proteins primarily bind to small RNAs, forming miRNA-induced silencing complexes that inhibit RNA degradation and suppress transcription. In humans, Ago2 proteins, functioning as active enzymes, interact with GW182 proteins to form GW bodies. These structures stabilize both intracellular and secreted miRNAs. These GW bodies then associate with DEAD-box helicase DDX6 to form processing bodies, aiding the silencing function of Ago2 protein.^66–69^ Han *et al.* examined the mechanisms of Ago2 ubiquitination clearance and miRNA degradation mechanisms. Their findings suggest that ZSWIM8, a cullin-RING ubiquitin ligase, mediates ubiquitin transfer to Ago2. This process triggers proteasomal degradation of miRNA-loaded Ago2, which consequently releases the associated miRNAs.^70^

Exosomes are not merely small vesicles with a specific function but are environmentally responsive structures widely distributed across organisms, including eukaryotes, prokaryotes, and viruses. They serve as vehicles for cells to transmit networked information and act as protective shells, analogous to “envelopes,” for transportation.^71^ For example, the functional properties of MC-EXOs depend on stimulation status. Exosomes from unstimulated murine MCs bind the high-affinity IgE receptor, reducing IgE levels and consequently inhibiting allergic inflammatory responses.^72^ This functional plasticity indicates that exosomes mediate diverse biological signals.

Exosomes originating from MCs secreted in response to immune antigen stimulation exert inflammatory effects on the gut. However, experiments have shown that exosomes can be used therapeutically for various diseases.^62,73,74^ For instance, exosomes derived from human adipose tissue mesenchymal stem cells can be used in cell-free therapy for atopic dermatitis. They inhibit the upregulation of IL-4, IL-31, IL-23, and TNF-α, exerting effects similar to corticosteroids, such as prednisolone.^73^ Li *et al.* found that exosomes from hypoxia-preconditioned hair follicle mesenchymal stem cells deliver miR-214-3p to modulate the PI3K/AKT/mTOR pathway. This action sustains mitochondrial dynamic stability, enhances autophagy in MODE-K cells and murine colitis models, and ameliorates ulcerative colitis.^61^ Compared to stem cell therapy, stem cell-derived exosomes offer advantages including lower tumorigenic potential, long-term storage capability, and absence of ethical concerns. Other studies demonstrate that miRNA-590-3p in M2 macrophage-derived exosomes targets and inhibits LATS1. This activates the YAP/β-catenin pathway, forming the YAP/β-catenin/TCF4 complex, which promotes IEC regeneration.^62^ Yang *et al.* proposed an autologous exosome therapy. They purified intestinal-derived exosomes isolated using a multistep sucrose gradient ultracentrifugation method from feces samples of healing-phase mouse models. They revealed that these exosomes exert an anti-inflammatory effect on DSS-treated mice.^74^

Furthermore, owing to their excellent drug delivery properties, exosomes exhibit significant promise for clinical applications. Examples include Patisiran and Ibudilast, which have been approved for treating neurological disorders.^75^ Ultracentrifugation suffers from low yield, the recent development of tangential flow filtration offers a cost-effective and more convenient approach for exosome isolation, thereby providing potential technical support for clinical translation.

TJs are crucial structures for cellular stability across various tissues, including the skin, blood vessels, blood–brain barrier, and intestinal mucosa. In an experiment examining the effects of *Akkermansia muciniphila* colonization on the intestine, the use of cAMP-responsive element-binding protein H inhibitor was found to suppress the expression of Claudin-8, which altered the intestinal permeability and inflammation.^33^ Wang *et al.* elucidated that in the pathogenesis of TNBS-induced IBD in rats, the IL-23/Th17 axis downregulates Claudin-8 expression through miRNA-223. This pathway compromises the intestinal mucosal barrier, where Claudin-8 serves as a downstream target of the IL-23 pathway.^32^ Li *et al.* demonstrated that HMC-EXOs interact with IECs through secretory pathways *in vitro* experiments. They identified that MC-EXOs deliver miR-223 to suppress Claudin-8 expression and increase intestinal epithelial permeability.^63^ However, the immune cell interactions in human ulcerative colitis are likely more complex *in vivo*.

We reviewed the precise interaction between exosomes and Claudin along with the functions of exosomes in disease development. We deem that MC-EXOs could be a promising diagnostic biomarker. Simultaneously, we could alleviate the severity of UC by blocking the pro-inflammatory effects of exosomes secreted by MCs. Consequently, the exosome secretion pathway in MC can emerge as a promising therapeutic target in UC. This review aims to provide new insights into the mechanisms underlying UC development and potentially contribute to improved diagnostic and therapeutic approaches for UC. However, future clinical diagnostic research on exosomes should incorporate multi-omics data and single-cell communication assays, providing the theoretical foundation for clinical translation through *in vivo* validation.

## 9. Methods

See the SI file.

## Author contributions

S. H. L. and HMX designed the study, summarised the literature, drafted the article and contributed equally to this paper; S. H. L. participated in the design of figures, data analysis and visualisation; Y. J. Z and H. L. H. planned and directed the project and interpreted the results. All authors read and approved the final manuscript.

## Conflicts of interest

The authors declare no competing financial interests.

## Abbreviations

UC: Ulcerative colitis
TJ: Tight junction
CLDN23: Claudin-23
MC-EXOs: Mast cell-derived exosomes
HMC-EXOs: Human mast cell-derived exosomes
MC-ILVs: Mast cell-derived intraluminal vesicles
IECs-EXOs: Intestinal epithelial cell derived exosomes
Ago-2: Argonaute 2
GSDMD: Gasdermin D
CagA: Cytotoxin-associated gene A protein
EMT: Epithelial-mesenchymal transition

## Supplementary Material

NA-OLF-D5NA00707K-s001

## Data Availability

This study did not generate any new data. The identifiers for the existing datasets are provided in detail in the Methods section of the supplementary files. The sc RNA-SEQ data used in this paper were obtained from the GEO database under accession number GSE214695. (Garrido-Trigo A., Corraliza A. M., Veny M., *et al.* Macrophage and neutrophil heterogeneity at single-cell spatial resolution in human inflammatory bowel disease [published correction appears in *Nat Commun.*, 2024 Jan 29, **15**(1), 857, DOI: https://doi.org/10.1038/s41467-024-45212-3]. *Nat Commun.*, 2023, **14**(1), 4506. Published 2023 Jul 26. DOI: https://doi.org/10.1038/s41467-023-40156-6). Exosome-related data in this article are from the EVmiRNA website: https://guolab.wchscu.cn/EVmiRNA. Other data (miRNA intersection data) have been placed in the SI. See DOI: https://doi.org/10.1039/d5na00707k.

## References

[cit1] Le Berre C., Honap S., Peyrin-Biroulet L. (2023). Ulcerative colitis. Lancet.

[cit2] Feuerstein J. D., Moss A. C., Farraye F. A. (2019). Ulcerative Colitis. Mayo Clin. Proc..

[cit3] Saez A., Gomez-Bris R., Herrero-Fernandez B., Mingorance C., Rius C., Gonzalez-Granado J. M. (2021). Innate Lymphoid Cells in Intestinal Homeostasis and Inflammatory Bowel Disease. Int. J. Mol. Sci..

[cit4] Ungaro R., Mehandru S., Allen P. B., Peyrin-Biroulet L., Colombel J. F. (2017). Ulcerative colitis. Lancet.

[cit5] Han G., Kim H., Jang H., Kim E. S., Kim S. H., Yang Y. (2024). Oral TNF-α siRNA delivery via milk-derived exosomes for effective treatment of inflammatory bowel disease. Bioact. Mater..

[cit6] Rashidi M., Bijari S., Khazaei A. H., Shojaei-Ghahrizjani F., Rezakhani L. (2022). The role of milk-derived exosomes in the treatment of diseases. Front. Genet..

[cit7] Mathivanan S., Fahner C. J., Reid G. E., Simpson R. J. (2012). ExoCarta 2012: database of exosomal proteins, RNA and lipids. Nucleic Acids Res..

[cit8] Wang L., Yu Z., Wan S., Wu F., Chen W., Zhang B., Lin D., Liu J., Xie H., Sun X., Wu Z. (2017). Exosomes Derived from Dendritic Cells Treated with Schistosoma japonicum Soluble Egg Antigen Attenuate DSS-Induced Colitis. Front. Pharmacol..

[cit9] Skotland T., Hessvik N. P., Sandvig K., Llorente A. (2019). Exosomal lipid composition and the role of ether lipids and phosphoinositides in exosome biology. J. Lipid Res..

[cit10] Gruenberg J. (2020). Life in the lumen: The multivesicular endosome. Traffic.

[cit11] Nguyen M. A., Karunakaran D., Geoffrion M., Cheng H. S., Tandoc K., Perisic Matic L., Hedin U., Maegdefessel L., Fish J. E., Rayner K. J. (2018). Extracellular Vesicles Secreted by Atherogenic Macrophages Transfer MicroRNA to Inhibit Cell Migration. Arterioscler., Thromb., Vasc. Biol..

[cit12] Mao F., Wu Y., Tang X., Kang J., Zhang B., Yan Y., Qian H., Zhang X., Xu W. (2017). Exosomes Derived from Human Umbilical Cord Mesenchymal Stem Cells Relieve Inflammatory Bowel Disease in Mice. BioMed Res. Int..

[cit13] Zhou Y., Zhang X. L., Lu S. T., Zhang N. Y., Zhang H. J., Zhang J., Zhang J. (2022). Human adipose-derived mesenchymal stem cells-derived exosomes encapsulated in pluronic F127 hydrogel promote wound healing and regeneration. Stem Cell Res. Ther..

[cit14] Wen Z., Li S., Liu Y., Liu X., Qiu H., Che Y., Bian L., Zhou M. (2025). An engineered M2 macrophage-derived exosomes-loaded electrospun biomimetic periosteum promotes cell recruitment, immunoregulation, and angiogenesis in bone regeneration. Bioact. Mater..

[cit15] Sun M., Wu Y., Chen Z., Zhang B., Liu X., Ouyang P., Chen P., Chen L., He Z., Han T., Li H., Sun J., Cai S., Luo Q. (2025). Chimeric exosomes-derived immunomodulator restoring lymph nodes microenvironment for sensitizing TNBC immunotherapy. Nat. Commun..

[cit16] Sobiepanek A., Kuryk Ł., Garofalo M., Kumar S., Baran J., Musolf P., Siebenhaar F., Fluhr J. W., Kobiela T., Plasenzotti R., Kuchler K., Staniszewska M. (2022). The Multifaceted Roles of Mast Cells in Immune Homeostasis, Infections and Cancers. Int. J. Mol. Sci..

[cit17] Ben S., Huang X., Shi Y., Xu Z., Xiao H. (2023). Change in cytokine profiles released by mast cells mediated by lung cancer-derived exosome activation may contribute to cancer-associated coagulation disorders. Cell Commun. Signaling.

[cit18] Savage A., Risquez C., Gomi K., Schreiner R., Borczuk A. C., Worgall S., Silver R. B. (2023). The mast cell exosome-fibroblast connection: A novel pro-fibrotic pathway. Front. Med..

[cit19] Wang Y., Chen A. (2022). Mast cell-derived exosomal miR-181a-5p modulated trophoblast cell viability, migration, and invasion via YY1/MMP-9 axis. J. Clin. Lab. Anal..

[cit20] McKenzie A. J., Hoshino D., Hong N. H., Cha D. J., Franklin J. L., Coffey R. J., Patton J. G., Weaver A. M. (2016). KRAS-MEK Signaling Controls Ago2 Sorting into Exosomes. Cell Rep..

[cit21] Mead B., Tomarev S. (2017). Bone Marrow-Derived Mesenchymal Stem Cells-Derived Exosomes Promote Survival of Retinal Ganglion Cells Through miRNA-Dependent Mechanisms. Stem Cells Transl. Med..

[cit22] Wang M., Guo J., Zhao Y. Q., Wang J. P. (2020). IL-21 mediates microRNA-423-5p/claudin-5 signal pathway and intestinal barrier function in inflammatory bowel disease. Aging.

[cit23] Wang C., Wu N., Pei B., Ma X., Yang W. (2023). Claudin and pancreatic cancer. Front. Oncol..

[cit24] Otani T., Nguyen T. P., Tokuda S., Sugihara K., Sugawara T., Furuse K., Miura T., Ebnet K., Furuse M. (2019). Claudins and JAM-A coordinately regulate tight junction formation and epithelial polarity. J. Cell Biol..

[cit25] Günzel D., Yu A. S. (2013). Claudins and the modulation of tight junction permeability. Physiol. Rev..

[cit26] Ahmad R., Kumar B., Thapa I., Talmon G. A., Salomon J., Ramer-Tait A. E., Bastola D. K., Dhawan P., Singh A. B. (2023). Loss of claudin-3 expression increases colitis risk by promoting Gut Dysbiosis. Gut Microbes.

[cit27] Barmeyer C., Fromm M., Schulzke J. D. (2017). Active and passive involvement of claudins in the pathophysiology of intestinal inflammatory diseases. Pflugers Arch..

[cit28] Stio M., Retico L., Annese V., Bonanomi A. G. (2016). Vitamin D regulates the tight-junction protein expression in active ulcerative colitis. Scand. J. Gastroenterol..

[cit29] Gong Y., Hou J. (2017). Claudins in barrier and transport function-the kidney. Pfluegers Arch..

[cit30] Čužić S., Antolić M., Ognjenović A., Stupin-Polančec D., Petrinić Grba A., Hrvačić B., Dominis Kramarić M., Musladin S., Požgaj L., Zlatar I., Polančec D., Aralica G., Banić M., Urek M., Mijandrušić Sinčić B., Čubranić A., Glojnarić I., Bosnar M., Eraković Haber V. (2021). Claudins: Beyond Tight Junctions in Human IBD and Murine Models. Front. Pharmacol.

[cit31] Awad K., Barmeyer C., Bojarski C., Nagel O., Lee I. M., Schweiger M. R., Schulzke J. D., Bücker R. (2023). Epithelial Barrier Dysfunction in Diarrhea-Predominant Irritable Bowel Syndrome (IBS-D) via Downregulation of Claudin-1. Cells.

[cit32] Wang H., Chao K., Ng S. C., Bai A. H., Yu Q., Yu J., Li M., Cui Y., Chen M., Hu J. F., Zhang S. (2016). Pro-inflammatory miR-223 mediates the cross-talk between the IL23 pathway and the intestinal barrier in inflammatory bowel disease. Genome Biol..

[cit33] Wade H., Pan K., Duan Q., Kaluzny S., Pandey E., Fatumoju L., Saraswathi V., Wu R., Harris E. N., Su Q. (2023). Akkermansia muciniphila and its membrane protein ameliorates intestinal inflammatory stress and promotes epithelial wound healing via CREBH and miR-143/145. J. Biomed. Sci..

[cit34] Chen E., Chuang L. S., Giri M., Villaverde N., Hsu N. Y., Sabic K., Joshowitz S., Gettler K., Nayar S., Chai Z., Alter I. L., Chasteau C. C., Korie U. M., Dzedzik S., Thin T. H., Jain A., Moscati A., Bongers G., Duerr R. H., Silverberg M. S., Brant S. R., Rioux J. D., Peter I., Schumm L. P., Haritunians T., McGovern D. P., Itan Y., Cho J. H. (2021). Inflamed Ulcerative Colitis Regions Associated With MRGPRX2-Mediated Mast Cell Degranulation and Cell Activation Modules, Defining a New Therapeutic Target. Gastroenterology.

[cit35] Yang L., He H., Guo X. K., Wang J., Wang W., Li D., Liang S., Shao F., Liu W., Hu X. (2024). Intraepithelial mast cells drive gasdermin C-mediated type 2 immunity. Immunity.

[cit36] Raya-Sandino A., Lozada-Soto K. M., Rajagopal N., Garcia-Hernandez V., Luissint A. C., Brazil J. C., Cui G., Koval M., Parkos C. A., Nangia S., Nusrat A. (2023). Claudin-23 reshapes epithelial tight junction architecture to regulate barrier function. Nat. Commun..

[cit37] Lu J., Liu D., Tan Y., Deng F., Li R. (2021). M1 Macrophage exosomes MiR-21a-5p aggravates inflammatory bowel disease through decreasing E-cadherin and subsequent ILC2 activation. J. Cell. Mol. Med..

[cit38] Xiao Y., Lian H., Zhong X. S., Krishnachaitanya S. S., Cong Y., Dashwood R. H., Savidge T. C., Powell D. W., Liu X., Li Q. (2022). Matrix metalloproteinase 7 contributes to intestinal barrier dysfunction by degrading tight junction protein Claudin-7. Front. Immunol..

[cit39] Kuo W. T., Odenwald M. A., Turner J. R., Zuo L. (2022). Tight junction proteins occludin and ZO-1 as regulators of epithelial proliferation and survival. Ann. N. Y. Acad. Sci..

[cit40] Kolchakova D., Moten D., Batsalova T., Dzhambazov B. (2021). Tight Junction Protein Claudin-12 Is Involved in Cell Migration during Metastasis. Biomolecules.

[cit41] Yang Z., Wu S., Fontana F., Li Y., Xiao W., Gao Z., Krudewig A., Affolter M., Belting H. G., Abdelilah-Seyfried S., Zhang J. (2021). The tight junction protein Claudin-5 limits endothelial cell motility. J. Cell Sci..

[cit42] Saeedi B. J., Kao D. J., Kitzenberg D. A., Dobrinskikh E., Schwisow K. D., Masterson J. C., Kendrick A. A., Kelly C. J., Bayless A. J., Kominsky D. J., Campbell E. L., Kuhn K. A., Furuta G. T., Colgan S. P., Glover L. E. (2015). HIF-dependent regulation of claudin-1 is central to intestinal epithelial tight junction integrity. Mol. Biol. Cell.

[cit43] Hou J., Renigunta A., Yang J., Waldegger S. (2010). Claudin-4 forms paracellular chloride channel in the kidney and requires claudin-8 for tight junction localization. Proc. Natl. Acad. Sci. U. S. A..

[cit44] Sun W., Wu W., Fang X., Ge X., Zhang Y., Han J., Guo X., Zhou L., Yang H. (2024). Disruption of pulmonary microvascular endothelial barrier by dysregulated claudin-8 and claudin-4: uncovered mechanisms in porcine reproductive and respiratory syndrome virus infection. Cell. Mol. Life Sci..

[cit45] Hering N. A., Andres S., Fromm A., van Tol E. A., Amasheh M., Mankertz J., Fromm M., Schulzke J. D. (2011). Transforming growth factor-β, a whey protein component, strengthens the intestinal barrier by upregulating claudin-4 in HT-29/B6 cells. J. Nutr..

[cit46] Sharafutdinov I., Esmaeili D. S., Harrer A., Tegtmeyer N., Sticht H., Backert S. (2020). Campylobacter jejuni Serine Protease HtrA Cleaves the Tight Junction Component Claudin-8. Front. Cell. Infect. Microbiol..

[cit47] Xu A. T., Lu J. T., Ran Z. H., Zheng Q. (2016). Exosome in intestinal mucosal immunity. J. Gastroenterol. Hepatol..

[cit48] Leoni G., Neumann P. A., Kamaly N., Quiros M., Nishio H., Jones H. R., Sumagin R., Hilgarth R. S., Alam A., Fredman G., Argyris I., Rijcken E., Kusters D., Reutelingsperger C., Perretti M., Parkos C. A., Farokhzad O. C., Neish A. S., Nusrat A. (2015). Annexin A1-containing extracellular vesicles and polymeric nanoparticles promote epithelial wound repair. J. Clin. Invest..

[cit49] Di Vincenzo F., Yadid Y., Petito V., Emoli V., Masi L., Gerovska D., Araúzo-Bravo M. J., Gasbarrini A., Regenberg B., Scaldaferri F. (2023). Circular and Circulating DNA in Inflammatory Bowel Disease: From Pathogenesis to Potential Molecular Therapies. Cells.

[cit50] Liu H., Tian Y., Xue C., Niu Q., Chen C., Yan X. (2022). Analysis of extracellular vesicle DNA at the single-vesicle level by nano-flow cytometry. J. Extracell. Vesicles.

[cit51] Song Y., Zhao Y., Ma Y., Wang Z., Rong L., Wang B., Zhang N. (2021). Biological functions of NLRP3 inflammasome: A therapeutic target in inflammatory bowel disease. Cytokine Growth Factor Rev..

[cit52] Li X., Ji L. J., Feng K. D., Huang H., Liang M. R., Cheng S. J., Meng X. D. (2024). Emerging role of exosomes in ulcerative colitis: Targeting NOD-like receptor family pyrin domain containing 3 inflammasome. World J. Gastroenterol..

[cit53] Bulek K., Zhao J., Liao Y., Rana N., Corridoni D., Antanaviciute A., Chen X., Wang H., Qian W., Miller-Little W. A., Swaidani S., Tang F., Willard B. B., McCrae K., Kang Z., Dubyak G. R., Cominelli F., Simmons A., Pizarro T. T., Li X. (2020). Epithelial-derived gasdermin D mediates nonlytic IL-1β release during experimental colitis. J. Clin. Invest..

[cit54] Guo Y., Xu C., Gong R., Hu T., Zhang X., Xie X., Chi J., Li H., Xia X., Liu X. (2022). Exosomal CagA from Helicobacter pylori aggravates intestinal epithelium barrier dysfunction in chronic colitis by facilitating Claudin-2 expression. Gut Pathogens.

[cit55] Liu K., Dou R., Yang C., Di Z., Shi D., Zhang C., Song J., Fang Y., Huang S., Xiang Z., Zhang W., Wang S., Xiong B. (2023). Exosome-transmitted miR-29a induces colorectal cancer metastasis by destroying the vascular endothelial barrier. Carcinogenesis.

[cit56] Kyuno D., Bauer N., Schnölzer M., Provaznik J., Ryschich E., Hackert T., Zöller M. (2019). Distinct Origin of Claudin7 in Early Tumor Endosomes Affects Exosome Assembly. Int. J. Biol. Sci..

[cit57] Ikeda C., Haga H., Makino N., Inuzuka T., Kurimoto A., Ueda T., Matsuda A., Kakizaki Y., Ishizawa T., Kobayashi T., Sugahara S., Tsunoda M., Suda K., Ueno Y. (2021). Utility of Claudin-3 in extracellular vesicles from human bile as biomarkers of cholangiocarcinoma. Sci. Rep..

[cit58] Omari S., Roded A., Eisenberg M., Ali H., Fukuda M., Galli S. J., Sagi-Eisenberg R. (2024). Mast cell secretory granule fusion with amphisomes coordinates their homotypic fusion and release of exosomes. Cell Rep..

[cit59] Fang X., Gao F., Zheng L., Xue F. S., Zhu T., Zheng X. (2024). Reduced microRNA-744 expression in mast cell-derived exosomes triggers epithelial cell ferroptosis in acute respiratory distress syndrome. Redox Biol..

[cit60] Li F., Wang Y., Lin L., Wang J., Xiao H., Li J., Peng X., Dai H., Li L. (2016). Mast Cell-Derived Exosomes Promote Th2 Cell Differentiation via OX40L-OX40 Ligation. J. Immunol. Res..

[cit61] Li N., Zhao L., Geng X., Liu J., Zhang X., Hu Y., Qi J., Chen H., Qiu J., Zhang X., Jin S. (2024). Stimulation by exosomes from hypoxia-preconditioned hair follicle mesenchymal stem cells facilitates mitophagy by inhibiting the PI3K/AKT/mTOR signaling pathway to alleviate ulcerative colitis. Theranostics.

[cit62] Deng F., Yan J., Lu J., Luo M., Xia P., Liu S., Wang X., Zhi F., Liu D. (2021). M2 Macrophage-Derived Exosomal miR-590-3p Attenuates DSS-Induced Mucosal Damage and Promotes Epithelial Repair via the LATS1/YAP/β-Catenin Signalling Axis. J. Crohn's Colitis.

[cit63] Li M., Zhao J., Cao M., Liu R., Chen G., Li S., Xie Y., Xie J., Cheng Y., Huang L., Su M., Xu Y., Zheng M., Zou K., Geng L., Xu W., Gong S. (2020). Mast cells-derived MiR-223 destroys intestinal barrier function by inhibition of CLDN8 expression in intestinal epithelial cells. Biol. Res..

[cit64] Vietri M., Radulovic M., Stenmark H. (2020). The many functions of ESCRTs. Nat. Rev. Mol. Cell Biol..

[cit65] Jeppesen D. K., Fenix A. M., Franklin J. L., Higginbotham J. N., Zhang Q., Zimmerman L. J., Liebler D. C., Ping J., Liu Q., Evans R., Fissell W. H., Patton J. G., Rome L. H., Burnette D. T., Coffey R. J. (2019). Reassessment of Exosome Composition. Cell.

[cit66] Zhai L., Wang L., Teng F., Zhou L., Zhang W., Xiao J., Liu Y., Deng W. (2016). Argonaute and Argonaute-Bound Small RNAs in Stem Cells. Int. J. Mol. Sci..

[cit67] Lv Z., Wei Y., Wang D., Zhang C. Y., Zen K., Li L. (2014). Argonaute 2 in cell-secreted microvesicles guides the function of secreted miRNAs in recipient cells. PLoS One.

[cit68] Liu J., Liu Z., Corey D. R. (2018). The Requirement for GW182 Scaffolding Protein Depends on Whether Argonaute Is Mediating Translation, Transcription, or Splicing. Biochemistry.

[cit69] Majerciak V., Zhou T., Kruhlak M. J., Zheng Z. M. (2023). RNA helicase DDX6 and scaffold protein GW182 in P-bodies promote biogenesis of stress granules. Nucleic Acids Res..

[cit70] Han J., LaVigne C. A., Jones B. T., Zhang H., Gillett F., Mendell J. T. (2020). A ubiquitin ligase mediates target-directed microRNA decay independently of tailing and trimming. Science.

[cit71] Kalluri R., LeBleu V. S. (2020). The biology, function, and biomedical applications of exosomes. Science.

[cit72] Lecce M., Molfetta R., Milito N. D., Santoni A., Paolini R. (2020). FcεRI Signaling in the Modulation of Allergic Response: Role of Mast Cell-Derived Exosomes. Int. J. Mol. Sci..

[cit73] Cho B. S., Kim J. O., Ha D. H., Yi Y. W. (2018). Exosomes derived from human adipose tissue-derived mesenchymal stem cells alleviate atopic dermatitis. Stem Cell Res. Ther..

[cit74] Yang C., Zhang M., Sung J., Wang L., Jung Y., Merlin D. (2020). Autologous Exosome Transfer: A New Personalised Treatment Concept to Prevent Colitis in a Murine Model. J. Crohn's Colitis.

[cit75] Chen Y. F., Luh F., Ho Y. S., Yen Y. (2024). Exosomes: a review of biologic function, diagnostic and targeted therapy applications, and clinical trials. J. Biomed. Sci..

