# Supplementary material for: Mast cell-derived exosomes and claudin regulation in ulcerative colitis: emerging insights and therapeutic potential
Source: Nanoscale Adv. 2025 Sep 10;7(21):6774–85. doi: 10.1039/d5na00707k (PMC12516517; doi:10.1039/d5na00707k)
Supplement: NA-007-D5NA00707K-s001 [file NA-007-D5NA00707K-s001.pdf]

Supplementary Information (SI) for Nanoscale Advances.
This journal is © The Royal Society of Chemistry 2025

Supplement File

Fig. S1

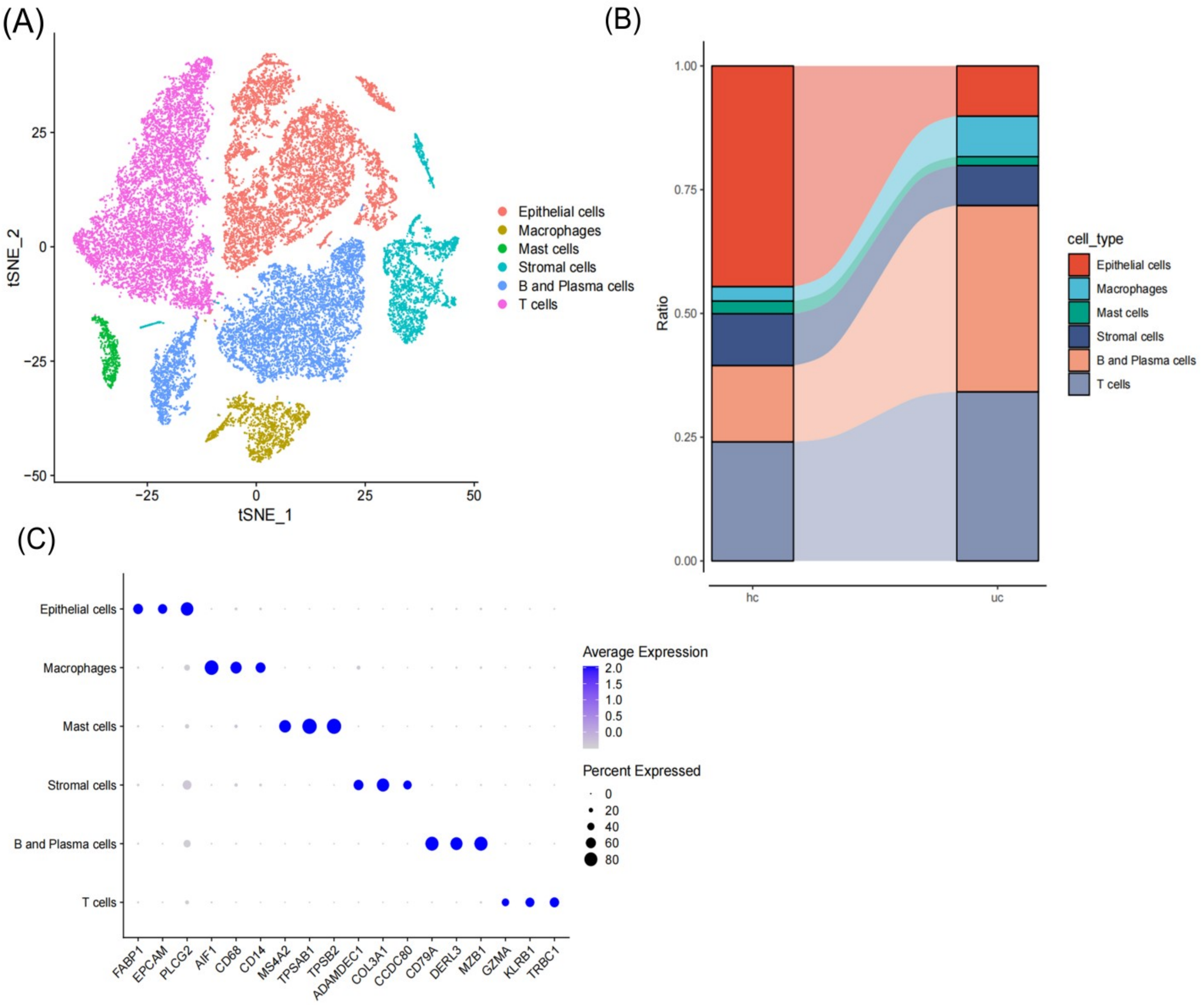

A). t-SNE plots of single-cell transcriptome sequencing of samples from healthy control (HC) and ulcerative colitis (UC) groups. B). Sankey plot of percentage change of each cell type in normal and ulcerative colitis groups. C). Marker genes of each cell type.

Methods

Single-cell RNA sequencing data analysis: Single-cell RNA data from GEO database GSE214695 included 6 healthy colon tissues and 6 active UC patient colon tissue samples. Following the original article's processing methods, low-quality cells were removed (criteria: nFeature_RNA > 100 & nFeature_RNA < 6000 & percent.mt < 65 & nCount_RNA > 500 & nCount_RNA < 25000 & percent.hb < 10). Each sample underwent normalization, variable features screening, PCA dimensionality reduction, batch effect removal using the Harmony package, and t-SNE clustering. Cells were annotated as intestinal epithelial cells, T cells (Th1/17), macrophages, B cells and plasma cells, mesenchymal cells, and mast cells. Cell-cell communication was analyzed using the cell chat package.

Bulk tissue microarray analysis: The GSE87466 dataset included 108 samples: 87 from UC patients and 21 from health controls. Differential gene expression analysis revealed 267 down-regulated genes. Gene Ontology (GO) enrichment analysis revealed that CLDN23, associated with epithelial tight junction signaling, was among these downregulated genes. Human mast cell (HMC)-derived exosomes were isolated from four leukemia patients (from the EVmiRNA database), and miRNA enrichment expression data was downloaded. HMC-miRNA expression types were cross-referenced with TARGETSCAN, MIRDB, and MIWALK databases to identify those predicted to bind the CLDN23 mRNA transcript (Figures 3C and 3D). Predicted miRNAs were prioritized based on high-confidence scores (total context++ score < -0.3 and aggregate Pct > 0.8; MIwalk score > 0.8; MIRDB score > 0.8). EVmiRNAs were filtered using data derived specifically from highly correlated human blood samples, excluding low-quality or insufficiently validated entries. miRNA expression levels were subsequently ranked. The HMC-miRNA satisfying all four intersections was hsa-miR-423-5p, and hsa-miR-486-5p matched the MIWALK intersection. However, hsa-miR-423-5p showed low expression in HMC-EXO, whereas hsa-miR-486-5p demonstrated the highest expression level among the candidates and was predicted to bind CLDN23. (core sequence-CCUGUAC).
